# Stuffing Teeth

**Published:** 1866-03

**Authors:** 


### Editorial.

### STUFFING TEETH.

We have within the past few months witnessed a large number of cases, in which operations upon the teeth, have been performed for arresting decay, commonly denominated filling, but in reality being nothing more than mere stuffing, incapable of excluding the fluids of the mouth and in many cases food.

We have been annoyed so much recently, by cases of this kind, coming from the hands of those, claiming to be first class dentists that we have determined to speak our mind on the subject; and we have also decided that in future, if there is not some improvement in this respect, one shall hint more definietly, than we now intend to do.

The following case will illustrate what we mean. To day a lady called, for treatment of her teeth for decay. Her teeth constitutionally good, and in good condition for successful operations at least so far as new decays are concerned. Her health was good, and she in all respects an admirable patient, for most successful operations upon her teeth, and in affluent circumstances. A year and a half ago, she called upon Dr.——— whom she was led to suppose by his assumption, and even by his general reputation, to be a thorough and successful operator; he filled or rather stuffed seven teeth, five with gold, and two with amalgam, none of the fillings, not even the amalgam, large, nearly all of them at points easy of access. Through every one of these gold fillings, an ordinary excavator would pass with a very moderate pressure, and the teeth decaying more or less about them all ; so that the cavities were from a third to a half larger then when filled. On the masticating surface of a superior molar, in the anterior depression, was a gold stuffing ; in a cavity in the posterior depression was an amalgam filling in contact with the gold, beneath a little bridge of enamel ; an excavator would pass down readily round both these fillings.

At first the lady avered she had no amalgam fillings in her teeth for Dr.——— would not put amalgam in her teeth, and was astonished when convinced by actual view ; she was but little less astonished when she saw the condition of the gold fillings, and the extent of the decay about them ; she indignatly remarked, take those things out, and fill my teeth as they should be. Now how stood the matter here ? this lady supposed these teeth that had been filled were secure, and, but for a timely examination would have remained under that impression till they would have been destroyed.

In this case, as in many others that have fallen under our observation, there was no excuse for this state of things, except the professional imbecility, or, depravity of the operator, the former we are inclined to believe more than the latter, for so far as the patient was concerned, there was every incentive to perform the best operations possible, and yet at the hands of this man, a poor miserable abortion was the result. We have become so annoyed, and so heart-sick with this kind of practice, that we are determined to probe it, till there is some improvement. Operations of this kind from the hands of those who make little pretentions, and are confessedly deficient in ability, do not involve a tithe of the danger accruing from such a case as we have given.

The man who assumes to be what he is not, and establishes a popular reputation, and thereby places the teeth of his patients in the sure road to ruin, and lulls them in security till that ruin is effected should receive the contempt of the profession and the scorn of the people.

